# Predictive values of immune indicators on respiratory failure in the early phase of COVID-19 due to Delta and precedent variants

**DOI:** 10.3389/fimmu.2023.1197436

**Published:** 2023-09-04

**Authors:** K. Nagaoka, H. Kawasuji, Y. Takegoshi, Y. Murai, M. Kaneda, K. Kimoto, S. Morimoto, H. Tani, H. Niimi, Y. Morinaga, Y. Yamamoto

**Affiliations:** ^1^ Department of Clinical Infectious Diseases, Toyama University Graduate School of Medicine and Pharmaceutical Sciences, Toyama, Japan; ^2^ Innovation Platform & Office for Precision Medicine, Nagasaki University Graduate School of Biomedical Sciences, Nagasaki, Japan; ^3^ Department of Virology, Toyama Institute of Health, Toyama, Japan; ^4^ Clinical Research Center for Infectious Diseases, Toyama University Graduate School of Medicine and Pharmaceutical Sciences, Toyama, Japan; ^5^ Department of Microbiology, Toyama University Graduate School of Medicine and Pharmaceutical Sciences, Toyama, Japan

**Keywords:** COVID-19, type I interferon, pneumonia, hypoxemia, interleukin-6, CXCL-10, humoral immune response

## Abstract

**Background:**

Immune response indicators in the early phase of COVID-19, including interferon and neutralizing responses against SARS-CoV-2, which predict hypoxemia remains unclear.

**Methods:**

This prospective observational study recruited patients hospitalized with COVID-19 (before emergence of omicron variant). As the immune indicators, we assessed the serum levels of IFN-I/III, IL-6, CXCL10 and VEGF, using an ELISA at within 5 days after the onset of symptoms, and serum neutralizing responses using a pseudovirus assay. We also assessed SARS-CoV-2 viral load by qPCR using nasal-swab specimens and serum, to assess the association of indicators and viral distribution.

**Results:**

The study enrolled 117 patients with COVID-19, of which 28 patients developed hypoxemia. None received vaccine before admission. Serum IFN-I levels (IFN-α and IFN-β), IL-6, CXCL10, LDH and CRP were significantly higher in patients who developed hypoxemia. A significant association with nasopharyngeal viral load was observed only for IFN-I. The serum levels of IFN-α, IL-6, CXCL10 were significantly associated with the presence of RNAemia. Multivariable analysis showed higher odds ratio of IFN-α, with cut-off value of 107 pg/ml, in regard to hypoxemia (Odds ratio [OR]=17.5; 95% confidence interval [CI], 4.7-85; p<0.001), compared to those of IL-6, >17.9 pg/ml (OR=10.5; 95% CI, 2.9-46; p<0.001).

**Conclusions:**

This study demonstrated that serum IFN-α levels in the early phase of SARS-CoV-2 infection strongly predict hypoxemic respiratory failure in a manner different from that of the other indicators including IL-6 or humoral immune response, and instead sensitively reflect innate immune response against SARS-CoV-2 invasion.

## Introduction

Coronavirus disease 2019 (COVID-19) is a highly transmissible infection caused by severe acute respiratory syndrome coronavirus 2 (SARS-CoV-2); the disease presentation and symptomology of COVID-19 ranges from asymptomatic to severe respiratory failure (1, 2). As increasing medical experience was acquired during the course of the pandemic, it has been recognized that severe COVID-19 is induced predominantly by complex immune regulation, rather than by cytokine storm syndrome (3, 4).

A recent meta-analysis conducted by Qin et al. (5), which evaluated 145 studies examining the association between immune-related indicators and COVID-19 prognosis, suggested that a combination of immunological, hematological, and biochemical parameters might be more sensitive in predicting disease severity and mortality following SARS-CoV-2 infection. As reported in previous studies, interleukin-6 (IL-6), the neutrophil-to-lymphocyte ratio (NLR), C-reactive protein (CRP) levels, and lactate dehydrogenase (LDH) levels have been determined to be the most representative parameters of SARS-CoV-2 infection; these findings have been repeatedly demonstrated in studies conducted in COVID-19 patients (6–8). We note that IL-6 is widely recognized as a pivotal cytokine in immune dysregulation, and also correlates with several important biomarkers (including C-X-C motif chemokine ligand 10 [CXCL10] and CRP) (9, 10).

To date, type I interferons (IFNs), which mainly consist of IFN-α and IFN-β, have emerged as crucial contributors to the innate immune response against SARS-CoV-2 infection (11, 12); interferons act as inhibitors of viral replication in infected cells and play a defensive role in uninfected cells. Although impairment of IFN-α and increased autoantibodies against IFN-α have been recognized as important contributors to the disease severity (11), the association between serum IFN-I levels and patient prognoses following SARS-CoV-2 infection remains unclear. Type III interferons (IFN-III), IL-29/IFN-λ1 and IL-28B/IFN-λ3, and which have received considerable attention as the predominant antiviral cytokines present at the mucosal barriers in the upper respiratory tract of SARS-CoV-2 infected patients, might potentiate the accurate prediction of coronavirus disease prognosis (13, 14).

Aside from those indicators that are representative of the innate immune response, humoral immune responses against SARS-CoV-2 infection have been considered the most robust form of immunity; humoral immune responses are induced by variable antibodies generated shortly after the onset of infection (15). Several studies have investigated the role of the humoral immune response in the early phase of SARS-CoV-2 infection in unvaccinated patients by measuring SARS-CoV-2 anti- receptor-binding domain titers or plasma/serum neutralizing responses using various methodologies (16–19). However, it remains unclear whether neutralizing activities against SARS-CoV-2 in the early phase of infection can predict favorable outcomes.

Herein, we sought to assess the potential of emerging immune indicators of SARS-CoV-2 as predictors of hypoxemia, in comparison with the biomarkers that are already in clinical use. In this study, we focused on the predictive value of immune indicators in the patients who had not received any vaccine against SARS-CoV-2 and infected by the Delta or precedent variants. The primary outcome of this study was to evaluate the association between indicators in the early phase of SARS-CoV-2 infection and the later development of hypoxemic respiratory failure. The secondary outcome of our investigation was to elucidate the pathophysiological implications of these indicators in relation to SARS-CoV-2 distribution.

## Materials and methods

### Study design

This study was conducted as part of the Toyama University COVID-19 Cohort Study, an investigator-initiated, prospective, single-center study, which was approved by the Ethical Review Board of the University of Toyama (R2019167).

The study period was between December 2020 and October 2021, which consisted of three major waves of the pandemic (before emergence of omicron) in Japan: the third wave (December to January 2021), the fourth wave (April to June 2021, which mainly occurred due to the Alpha variant), and the fifth wave (July to October 2021, which mainly occurred due to the Delta variant). Nasal specimens for reverse transcription quantitative polymerase chain reaction (RT-qPCR) were collected and chest CT was performed at admission; moreover, serum samples were stored frozen at -80°C after each laboratory examination. Written informed consent was obtained from all patients.

### Study participants and study protocol

The inclusion criteria were as follows: 1) men or women aged 18 years or older, who were diagnosed with COVID-19 based on the findings of RT-qPCR assays. 2) patients hospitalized at Toyama University Hospital (Toyama, Japan) between December 2020 and October 2021, and 3) patients with a first blood sample collected within five days after symptom onset.

Since the large population with COVID-19 had not received vaccine against SAR-CoV-2 during the study period, we excluded the following from the present study; patients who had received a vaccine or SARS-CoV-2 antibody treatment, or those who participated in another clinical trial.

Clinical data on patient were collected from patients’ medical charts. COVID-19 pneumonia was confirmed by trained pulmonary radiologists (KN and YY), when a newly developed inflammatory lesion was detected by chest CT performed on admission, according to the previous reports (20, 21). Patients without inflammatory lesions were confirmed to be negative for COVID-19 pneumonia.

Hypoxemia requiring oxygen therapy was defined according to a blood oxygen saturation (SpO_2_) level of ≤93% at rest on room air. This is a universally accepted criterion for the initiation of oxygen therapy for COVID-19 (22). Patients were excluded from further analysis, if had received oxygen therapy for hypoxemia (SpO_2_ ≤93% at rest) or had received any anti-viral or immunomodulate therapy at the time of admission.

### Blood samples

The stored blood serum samples of the patients were used for cytokine and RNAemia measurements as described below. Only serum collected within 5 days after symptom onset was used for the analysis.

### Cytokine measurement

Serum cytokines and chemokines (IFN-α, IFN-β, IFN-λ1 [IL-29], IFN-λ3 [IL-28-B], IL-6, CXCL10 and vascular endothelial growth factor [VEGF]) were measured using commercially available enzyme-linked immunosorbent assay (ELISA) kits, according to the manufacturers’ instructions (see **Supplemental Data**; **Table S1**). VEGF, which was also known as potential predictor for prognosis of COVID-19 (23), was included as a comparator indicator. If an analyte signal was below the background signal, it was set to 0; if the signal was detectable but below the manufacturer’s lower limit of quantification, it was set to the lower limit of detection. Each lower limit of detection was as follows; IFN-α for 0.43 pg/mL, IFN-β for 1.2 pg/mL, IFN-λ1 for 2.0 pg/mL, IFN-λ3 for 2.1 pg/mL, IL-6 for 1.2 pg/mL, CXCL10 for 13.4 pg/mL.

### RT-qPCR

RT-qPCR (for detecting SARS-CoV-2) was performed as previously described in a study conducted at our hospital (24). The detection limit was approximately 0.4 copies/μL (2 copies/5 μL). RNAemia was determined when SARS-CoV-2 was detectable in blood serum specimens.

The presence of mutation on SARS-CoV-2 was examined with the screening PCR tests, which was conducted as administrative tests at Toyama institution of health (Toyama, Japan), during the fourth and fifth wave. The presence of N501Y mutation (suspected as alpha variant, if positive) was examined with patients in the fourth wave, using Primer/Probe N501Y (Takara Bio Inc., Shiga, Japan). The presence of L452R (suspected as delta variant, if positive) was examined with patients in the fifth wave, using Primer/Probe L452R Ver.2 (Takara Bio Inc.).

### Pseudo-virus neutralization assay

The neutralizing activity of human serum against pseudo-viruses was measured using the high-throughput chemiluminescent reduction neutralizing test (htCRNT), as previously described (25). The values of samples without the pseudo-virus and those with the pseudo-virus but without serum were defined as 0% and 100% infection (100% and 0% inhibition), respectively.

In order to measure neutralizing activity (NT) against the infected variant of each pandemic wave, we used three pseudo-viruses with expression plasmids for the truncated S protein of SARS-CoV-2; pCAG-SARS-CoV-2 S (Wuhan; wild-type [WT]), pCAGG-pm3-SARS2-Shu-d19-B1.1.7 (Alpha-derived variant), and pCAGG-pm3-SARS2-Shu-d19-B1.617.2 (Delta-derived variant). For all patients, NT was evaluated against the WT strain using a pseudo-virus with the truncated S protein of the WT strain. In addition, NT against the infected variant was measured for the fourth and fifth pandemic waves; NT against alpha variant for the fourth wave, and NT against delta variant for the fifth wave. We defined the NT against each SARS-CoV-2 variant of that pandemic wave as the “adjusted NT.”

### Statistical analysis

A summary of the participants’ medical and demographic characteristics was expressed using medians (interquartile ranges) or numbers (percentages). Differences between the two groups were tested using the Mann–Whitney test or Fisher’s exact test. The Mann–Whitney U test with Bonferroni correction was used to compare nominal variables among three groups.

Receiver operating characteristic (ROC) curves and the respective areas under the ROC curve (AUC) were generated using GraphPad Prism 9 software (GraphPad Software, San Diego, CA, USA). The cut-off value for the prediction of hypoxemia was determined using the nearest point relative to the left corner of each ROC curve.

The association between each pair of biomarkers and nasopharyngeal viral load was determined using Spearman’s rho correlation coefficient.

The association of each biomarker with hypoxemic respiratory failure was estimated as an adjusted odds ratio (OR) calculated via a logistic regression model adjusting for potential confounders that were determined based on clinical considerations (age, sex, body mass index [BMI], and patients’ present history of hypertension/diabetes mellitus). These variables were dichotomized as above or below the cut-off value calculated using ROC curve analysis. The size of the tests was set to 0.05 and statistical significance was set to p <0.05. R statistical software (v.4.1.018; The R Project for Statistical Computing, Vienna, Austria) and GraphPad Prism software (v.9.0) were used for the statistical analyses.

## Results

### Study participants

The clinical characteristics, microbiological findings, treatments, and outcomes of the 117 patients included in this study are summarized in **Table 1**. We note that a portion of the study population was included in previous experimental reports investigating the clinical implication of IFN-I (n = 50) (26), and the effect of monoclonal antibody treatment (n = 28) (27). A total of 28 patients in the entire cohort developed hypoxemia, while 89 did not. Age, underlying disease (hypertension, diabetes mellitus), and BMI were significantly different between patients with COVID-19 who did or did not develop hypoxemia. All enrolled patients survived COVID-19 for at least 30 days after symptom onset.

**Table 1:** Clinical features, microbiological findings of patients in the study.

	Total (n=117)	Hypoxemia required oxygen therapy		P value
		Positive (n=28)	Negative (n=89)	
Age, years	46 [31-54]	57 [51-64]	38 [26-51]	<0.001
Sex; male/female	71/46	23/5	48/41	0.008
Pandemic period				
Third wave	39 (33%)	1 (3%)	38 (43%)	—
Fourth wave	50 (43%)	17 (61%)	33 (37%)	—
Fifth wave	28 (24%)	10 (36%)	18 (20%)	—
Underlying disease				
None	63 (54%)	9 (32%)	54 (61%)	0.010
Hypertension	21 (18%)	12 (43%)	9 (10%)	<0.001
Diabetes mellitus	8 (7%)	5 (18%)	3 (3%)	0.019
Body mass index	22.8 [20.8-25.5]	25.3 [23.4-27.6]	21.6 [20.5-24.4]	<0.001
Initial nasopharyngeal-viral load (log copies/μL)	4.8 [3.8-5.5]	5.2 [4.0-5.6]	4.7 [3.8-5.4]	0.185
RNAemia	31 (26%)	16 (57%)	15 (17%)	<0.001
Treatment				
Remdesivir	29 (25%)	28 (100%)	1 (1%)	—
Dexamethasone	31 (26%)	28 (100%)	3 (3%)	—
Heparin	31 (26%)	28 (100%)	3 (3%)	—
Nasal High Flow	4 (3%)	4 (14%)	—	—
IPPV	2 (2%)	2 (7%)	—	—
30 days-mortality	0 (0%)	0 (0%)	0 (0%)	—

Continuous variables are reported as median [interquartile range (IQR) 25-75]. Categorical variables are reported as number (percentages).

‘—’ indicates that the data were not applicable for evaluation nor comparison.

### Serum biomarkers and the development of hypoxemic respiratory failure

**Figure 1:**
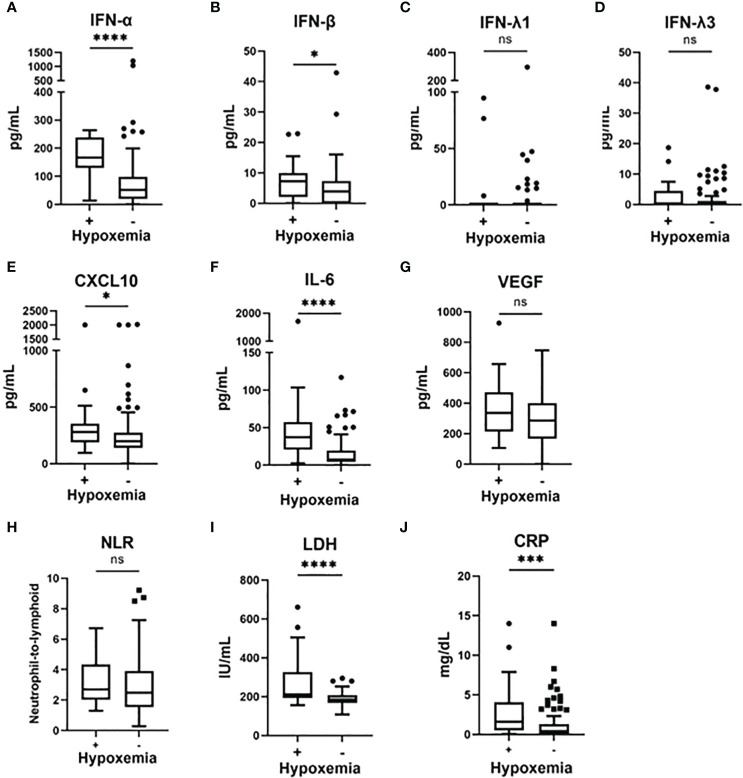
Serum biomarker levels in the early phase of SARS-CoV-2 infection and associations with the development of hypoxemic respiratory failure. Serum biomarker levels in the early phase of SARS-CoV-2 infection and associations with the development of hypoxemic respiratory failure: **(A)** IFN-α, **(B)** IFN-β, **(C)** IFN-λ1, **(D)** IFN-λ3, **(E)** CXCL10, **(F)** IL-6, **(G)** VEGF, **(H)** NLR, **(I)** LDH, and **(J)** CRP. Each biomarker level was evaluated at hospital admission (within five days after symptom onset), without hypoxemic respiratory failure being present at that time. Data are presented using Tukey box-plots as well as using individual values. ^*^p <0.05; ^***^p <0.001; ^****^p <0.0001; ns, not significant.

The results of the biomarker-level analyses are summarized in **Figures 1A–J**. Because the analyte signals for CXCL10 were higher than the detectable range in four patients (too strong analyte signal to be detected), we set the highest value of CXCL10 to 2,000 pg/mL in further analyses. We found that IFN-α, IFN-β, IL-6, CXCL10, LDH, and CRP levels were significantly higher in patients who later developed hypoxemia than in those who did not.

### Serum neutralizing activities against SARS-CoV-2 strains

**Figure 2:**
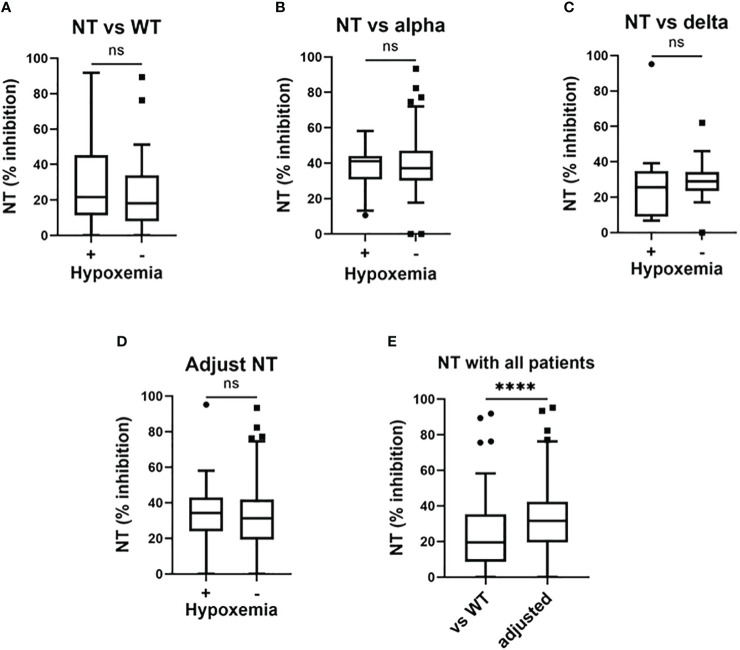
Serum neutralizing activities in the early phase of SARS-CoV-2 infection and associations with the development of hypoxemic respiratory failure. Serum neutralizing activities (NT; % inhibition) in the early phase of SARS-CoV-2 infection (i.e., within five days after symptom onset) and associations with the development of hypoxemic respiratory failure: **(A)** NT against the wild-type (WT) strain in patients enrolled in the third, fourth, and fifth pandemic waves (n = 117); **(B)** NT against the Alpha variant in patients enrolled in the fourth pandemic wave (n = 50); **(C)** NT against the Delta variant in patients enrolled in the fifth pandemic wave (n = 28); **(D)** adjusted NT values in patients enrolled in the third, fourth, and fifth pandemic waves (n = 117); **(E)** NT against the WT strain and adjusted NT values in patients enrolled in the third, fourth, and fifth study waves. Each level was evaluated at hospital admission (within five days after symptom onset), without hypoxemic respiratory failure being present at that time. Adjusted NT values were significantly higher than those of NT against the WT strain. ^****^p <0.0001; ns, not significant.

Our results in regard to NT against SARS-CoV-2 strains are summarized in **Figure 2**. With patients in the third, fourth, and fifth wave, NT against the WT strain were not significantly different between those who developed hypoxemia and those who did not (**Figure 2A**). Similarly, the adjusted NT were not significantly different between patients who developed hypoxemia and those who did not develop hypoxemia (**Figures 2B–D**).

The presence of N501Y mutation was confirmed in 94% of the fourth wave, and the presence of L452R was confirmed in 82% of the fifth wave. Whereas, neither mutation was unknown in several cases; 3 out of 50 patients in the fourth wave (none developed hypoxemia), and 5 out of 28 the fifth wave (3 developed hypoxemia).

NT values against the WT strain in the whole cohort were significantly lower than the findings for adjusted NT (**Figure 2E**).

In the measurement of NT against each variant, we simultaneously examined time-dependent changes in NT in several patients to confirm whether NT values could consistently reflect the neutralizing response against SARS-CoV-2 infection. As shown in **Figure S1**, NT against each strain increased until two weeks after symptom onset, which was confirmed in 14/14 patients with available serum.

### Biomarker levels and SARS-CoV-2 viral load

**Figure 3:**
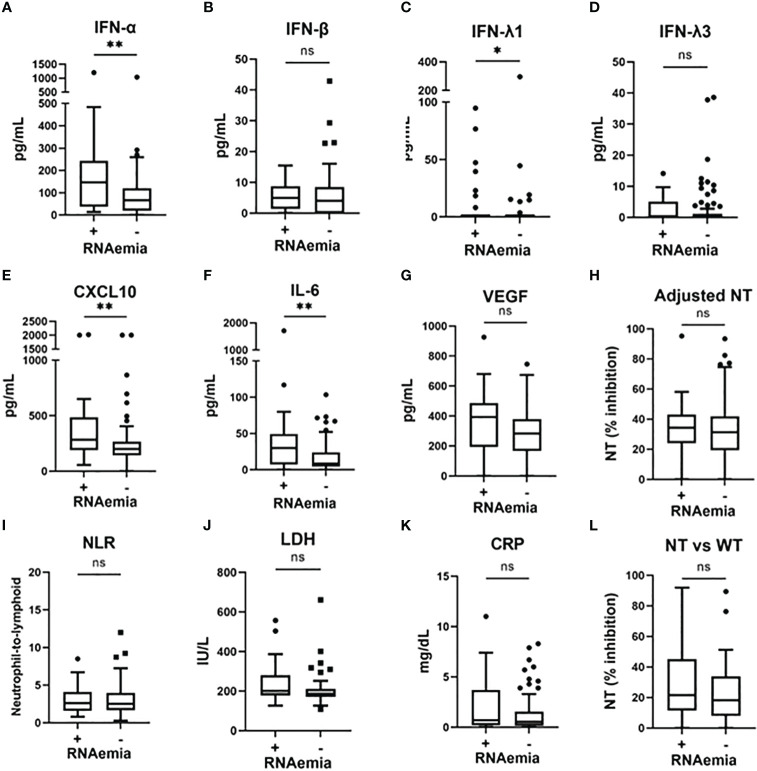
Serum biomarker levels in the early phase of SARS-CoV-2 infection and associations with the presence of RNAemia. Serum biomarker levels in the early phase of SARS-CoV-2 infection and associations with the presence of RNAemia: **(A)** IFN-α, **(B)** IFN-β, **(C)** IFN-λ1, **(D)** IFN-λ3, **(E)** CXCL10, **(F)** IL-6, **(G)** VEGF, **(H)** adjusted NT, **(I)** NLR, **(J)** LDH, **(K)** CRP, and **(L)** NT vs WT. Each level was evaluated at a time point at hospital admission (within five days after symptom onset). Data are presented using Tukey box-plots and individual values. ^*^p <0.05, ^**^p <0.005; ns, not significant. NT, neutralizing activities (% inhibition); NT vs WT, neutralizing activities against the wild-type strain.

To examine the association between microbiological findings and biomarker levels, we measured the viral load in nasal-swab specimens and in serum. We could not assess the nasal viral load in five patients (all did not develop hypoxemia), because we could not collect the nasal-swab specimen for this observational study. As shown in **Figure S2**, the levels of IFN-α and IFN-β, as well as evaluations NT against the WT strain, significantly correlated with the SARS-CoV-2 viral load in nasal-swab specimens. A stronger correlation was observed with IFN-β than with IFN-α. Moreover, as shown in **Figure 3**, IFN-α, IFN-λ1, CXCL10, and IL-6 levels were significantly higher in patients with RNAemia than in those without RNAemia.

### Association between biomarker levels and the presence of pneumonia

We analyzed the association between various biomarkers and the presence of pneumonia. As shown in **Figure S3**, in addition to the biomarkers that were associated with the development of hypoxemia (IFN-α, IL-6, CXCL10, LDH, and CRP), VEGF, NLR, and adjusted NT levels were significantly higher in patients with pneumonia than in those without pneumonia.

### Predictors of hypoxemic respiratory failure due to SARS-CoV-2 infection

**Figure 4:**
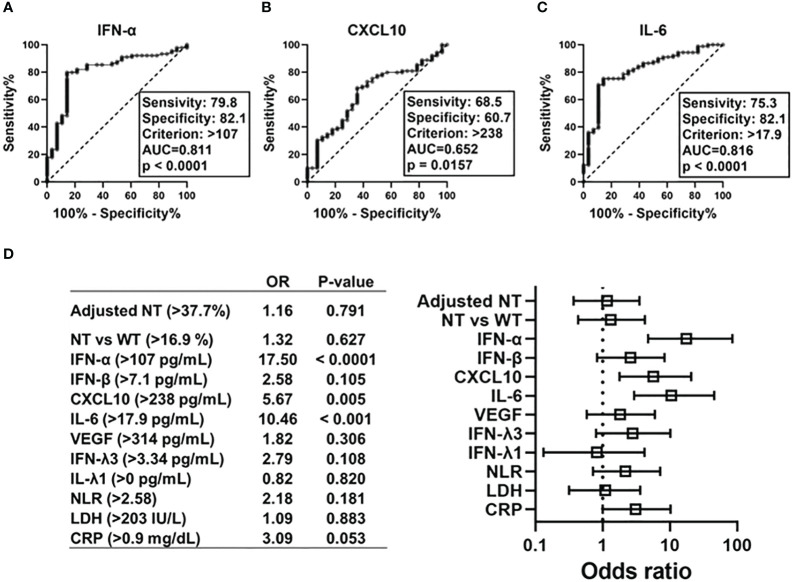
Predictive value of each immune indicators on development of hypoxemic respiratory failure in the early phase of SARS-CoV-2 infection. ROC curves and AUCs for biomarker levels in patients with SARS-CoV-2 infections in regard to diagnostic values indicating respiratory failure: **(A)** IFN-α, **(B)** CXCL10, and **(C)** IL-6. Forest plots representing odds ratios for each biomarker in regard to the development of hypoxemia at an early phase of SARS-CoV-2 infection (within five days after symptom onset) **(D)**. Each variables were dichotomized as above or below the cut-off value calculated using ROC curve analysis. Each odds ratio (OR) is adjusted for age, sex, body mass index, and history of hypertension/diabetes mellitus. AUC, area under ROC curve; NT, neutralizing activities (% inhibition); NT vs WT, neutralizing activities against the wild-type strain; ROC, receiver operating characteristic.

**Figure 5:**
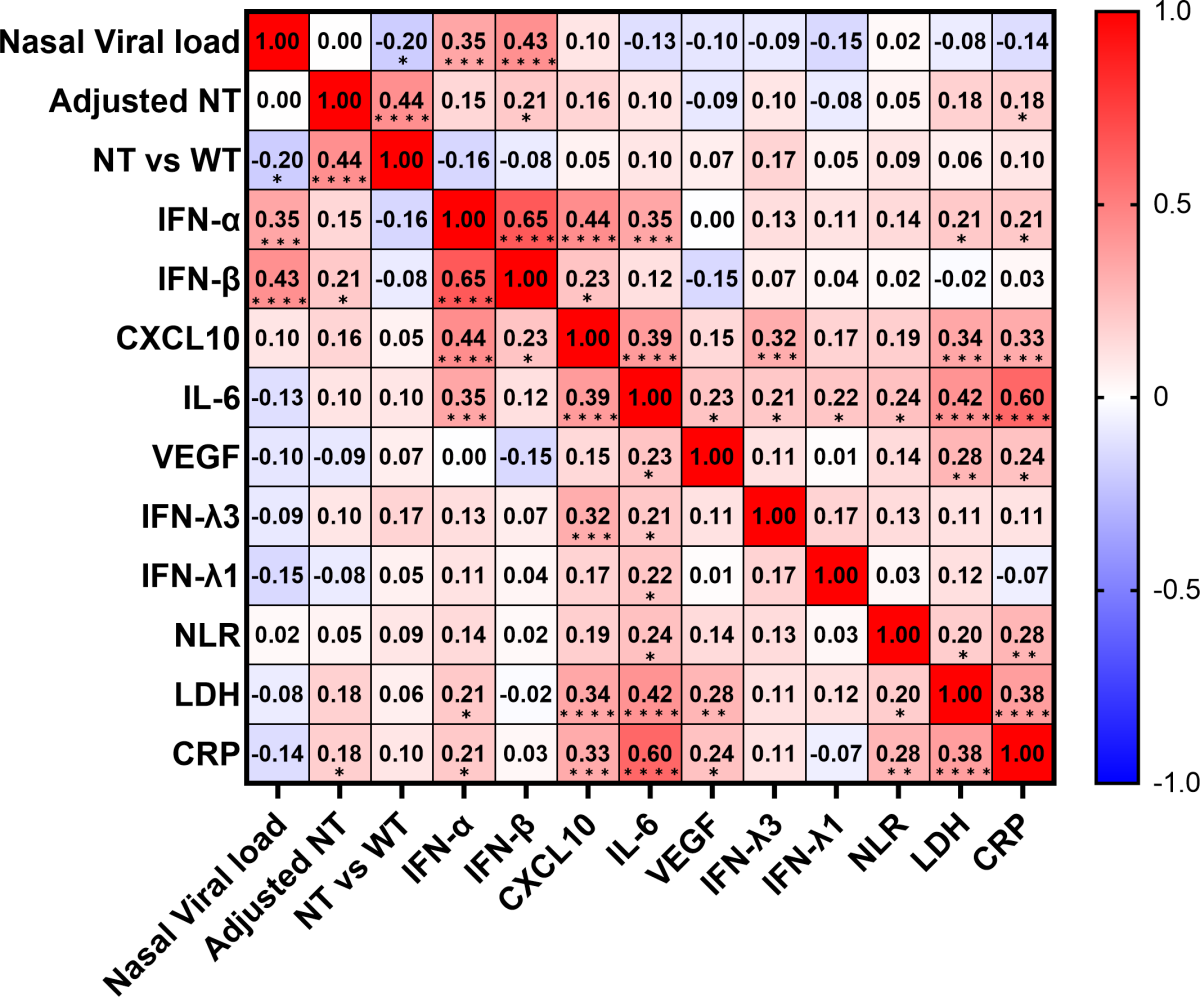
Correlation matrix of biomarkers in patients with SARS-CoV-2 infection in the early phase. Correlation matrix of biomarkers in patients with SARS-CoV-2 infection in the early phase (within five days after symptom onset). Results are presented as a correlation matrix. Spearman correlation coefficients are plotted. Cells were colored according to the strength and trend of correlations (shades of red = positive correlations, shades of blue = negative correlations). ^*^p <0.05; ^**^p <0.005; ^***^p<0.001; ^****^p<0.0001. NT, neutralizing activities (% inhibition); NT vs WT, neutralizing activities against the wild-type strain.

To determine the predictors of hypoxemic respiratory failure occurring due to SARS-CoV-2 infection and its respective cut-off values, we conducted an ROC analysis for each biomarker (**Figures 4A–C**; **Figure S4**). AUCs were higher than >0.8 for IFN-α and IL-6. Multivariate regression analysis showed that serum IFN-α levels higher than the cut-off value of 107 pg/mL showed the highest OR for hypoxemic respiratory failure, demonstrating a greater than two-fold stronger association as compared with IL-6 and CXCL10 (**Figure 4D**). Among the evaluated biomarkers, IFN-β, CXCL-10, IL-6, LDH, and CRP levels significantly and directly correlated with serum IFN-α levels (**Figure 5**). In contrast, IL-6 and CXCL10 levels correlated more strongly and significantly correlated with LDH and CRP levels. Notably, IFN-β levels significantly correlated with adjusted NT values and with IFN-α levels.

## Discussion

This study demonstrated that serum IFN-α levels present a higher OR in regard to the development of hypoxemia in the early phase of COVID-19 than those of other biomarkers, including IL-6. The distinct features of IFN-α in relation to other IFNs and cytokines, the distribution of the virus, and findings in regard to neutralization activity support our preliminary conclusion (26) that IFN-α could be a unique and strong predictor of hypoxemia occurring due to SARS-CoV-2 infection. Also, these strongly support that IFN-α play critical role in the early phase of COVID-19 due to Delta and the precedent variants.

In our study, IL-6 was one of the most significant predictors of hypoxemia due to SARS-CoV-2 infection; hypoxemia was also closely related to CXCL10, LDH, and CRP levels. The mean level of IL-6 in patients with hypoxemia enrolled in our study, 37.3 pg/mL [20.8-55.5], was similar to that reported in a recent study (10).

Among the indicators measured in this study, IFN-I, IFN-α and IFN-β, significantly correlated with nasopharyngeal viral load in the early phase of SARS-CoV-2 infection, indicating that IFN-I sensitively interacts with viral replication rather than with other biomarkers. Moreover, we confirmed that IFN-α was significantly associated with RNAemia, consistent with the findings of our previous study (26). The strong association between IFN-α and RNAemia detected herein may explain why IFN-α was the most significant predictor of hypoxemia in the current study.

Previous studies have suggested that an impaired IFN-I response and lower serum IFN-α levels could be hallmarks of severe COVID-19 (11, 12). To date, a few studies have investigated IFN-α levels in the early phase of SARS-CoV-2 infection (i.e., within five days after symptom onset), and these studies detected a range of serum IFN-α levels (28, 29). In the current study, we confirmed that serum IFN-α levels with a cut-off level of 107 pg/mL were significantly associated with the development of hypoxemia in patients enrolled during different pandemic periods. In contrast, we also found that a few of our enrolled patients developed hypoxemia with low IFN-α levels (<100 pg/mL), namely 1/17 patients (5.9%) who developed hypoxemia in the fourth wave and 2/10 patients (20.0%) who developed hypoxemia in the fifth wave. These incidents were similar to those of sub-phenotypes with IFN-I autoantibodies (˜20%) (30). With COVID-19 in the early pandemic period (March-May 2020), several studies demonstrated the association between lower IFN-α and fatal COVID-19 (31, 32). In the present study, six patients required mechanical ventilation or nasal high flow, and one of those presented lower IFN-α levels (19.5 pg/mL). This supports the possibility that sub-phenotype may exist, which follow fatal course with lower IFN-α levels at the early phase of COVID-19. The caution might be necessary in regard to specifying the sub-phenotype, in the application of serum IFN-α levels as a clinical predictor of hypoxemia due to SARS-CoV-2 infection.

In this study, we measured serum neutralizing responses against the SARS-CoV-2 WT strain and against each coronavirus variant using the pseudo-virus assay, and explored the relationship between biomarkers and prognosis following SARS-CoV-2 infection. As shown in **Figure S1**, we confirmed that all neutralizing ht-CRNT values were elevated over 90% until two weeks after symptom onset, which supports the speculation that the lower neutralizing value in the early phase of SARS-CoV-2 infection consistently reflects lower humoral immune responses against SARS-CoV-2. Notably, the assessment of serum neutralizing activities in the early phase of SARS-CoV-2 infection revealed that the adjusted neutralizing value was significantly associated with the presence of pneumonia (**Figure S3**), but not with the development of hypoxemia or the presence of RNAemia. This was partly consistent with a previous study conducted by Park et al., which found a significant correlation between neutralizing titers and chest radiography scores in 40 patients with COVID-19 (18).

It is possible that a passive immune response could be induced earlier in patients who develop pneumonia than in those who do not. Among the indicators measured in this study, IFN-β and CRP levels significantly correlated with adjusted NT, which strongly suggests interactions between IFN-β, CRP, and the activation of the humoral immunity response against SARS-CoV-2. Additional studies are necessary to investigate the detailed pathophysiology of neutralization as well as associated biomarkers. According to the results of this study, we suggest that the neutralizing value in the early phase of SARS-CoV-2 infection might be poorly associated with the prediction of respiratory failure.

The present study has several limitations. First, the single-center observational study design of the present study, along with a modest sample size, may have resulted in selection bias. Second, we validated IFN and cytokine levels using serum samples that were not strictly stored immediately after drawing (these samples were instead immediately stored in a 4°C freezer and were then transferred to a -80°C deep freezer). Third, we could not identify the causative strain in the third pandemic wave and a part of the fourth and fifth wave, because genetic identification of the epidemic strain was not available. Therefore, our results in regard to adjusted NT did not completely reflect NT against the infected strain. Fourth, we did not assess the predictive value of immune indicators in patients with COVID-19 by Omicron variant, in this study. The Omicron variant rapidly outcompeted Delta variant by late 2021, and has dominated the pandemic until today (33). In order to promote IFN-α as the predictive biomarker of COVID-19 in clinical use, further assessment in patients with COVID-19 by Omicron variant would be necessary. However, considering our consistent results and the detected associations between IFN, the major cytokine evaluated herein, and NT, we believe that these limitations were not likely to have meaningfully affected our findings, which elucidated the critical part of immune dynamics in COVID-19 without vaccination, due to the precedent virulent variants other than Omicron variant.

In conclusion, we demonstrated that serum IFN-α levels strongly predict hypoxemic respiratory failure in the early phases of COVID-19 by the Delta and the precedent variants, as do IL-6 levels. We suggest that early elevation of serum IFN-α levels may reflect an innate immune response against SARS-CoV-2 systemic invasion, which could be a novel indicator of hypoxemic respiratory failure. These findings highlight the most important feature of immune indicators in COVID-19 during the pandemic period before emergence of Omicron variant, not only as a highly potential predictive factor of hypoxemic respiratory failure but also as a clue to understanding the pathophysiology of COVID-19 due to the current and future variants.

## Data availability statement

The original contributions presented in the study are included in the article/**Supplementary Material**. Further inquiries can be directed to the corresponding author.

## Ethics statement

The studies involving humans were approved by The Ethical Review Board of the University of Toyama. The studies were conducted in accordance with the local legislation and institutional requirements. The participants provided their written informed consent to participate in this study.

## Author contributions

Conceptualization, KN; Methodology, KN, HK, HT; Software, KN; Validation, KN, HK, YMo; Formal Analysis, KN; Statistical Analyses, KN, SM; Investigation, KN and HK; Resources, KN; Data Curation, KN, HK, YMu, MK, KK, HN, YMo; Writing – Original Draft Preparation, KN; Writing – Review & Editing, KN, YMo, and YY; Visualization, KN; Supervision, YY; Project Administration, KN and YY; Funding Acquisition, YY. All authors contributed to the article and approved the submitted version.
