# Supplementary material for: Predictive values of immune indicators on respiratory failure in the early phase of COVID-19 due to Delta and precedent variants
Source: Front Immunol. 2023 Sep 4;14:1197436. doi: 10.3389/fimmu.2023.1197436 (PMC10507327; doi:10.3389/fimmu.2023.1197436)
Supplement: Supplementary file 1 [file DataSheet_1.docx]

Supplementary Table and Figure

# Predictive values of immune indicators on development of hypoxemic respiratory failure in the early phase of SARS-CoV-2 infection

**Table S1. The list of ELISA kit used in this study.**

| Immune indicators | ELISA kit | Manufacturer |
| --- | --- | --- |
| IFN-α | VeriKine-HS Human IFN Alpha All Subtype ELISA Kit | PBL Assay Science, Piscataway, NJ, USA |
| IFN-β | VeriKine-HS Human IFN Beta Serum ELISA Kit | PBL Assay Science, Piscataway, NJ, USA |
| CXCL10 | Human CXCL10/IP-10 ELISA Kit | Proteintech, Rosemont, IL, USA |
| IL-6 | AuthentiKine™ Human IL-6 ELISA Kit | Proteintech, Rosemont, IL, USA |
| VEGF | AuthentiKine™ Human VEGF ELISA Kit | Proteintech, Rosemont, IL, USA |
| IFN-λ1/IL-29 | IL-29 Human ELISA Kit | Invitrogen, Waltham, MA, USA |
| IFN-λ3/IL-28B | AuthentiKine™ Human IL-28B ELISA Kit | Proteintech, Rosemont, IL, USA |

**Figure Supple 1**


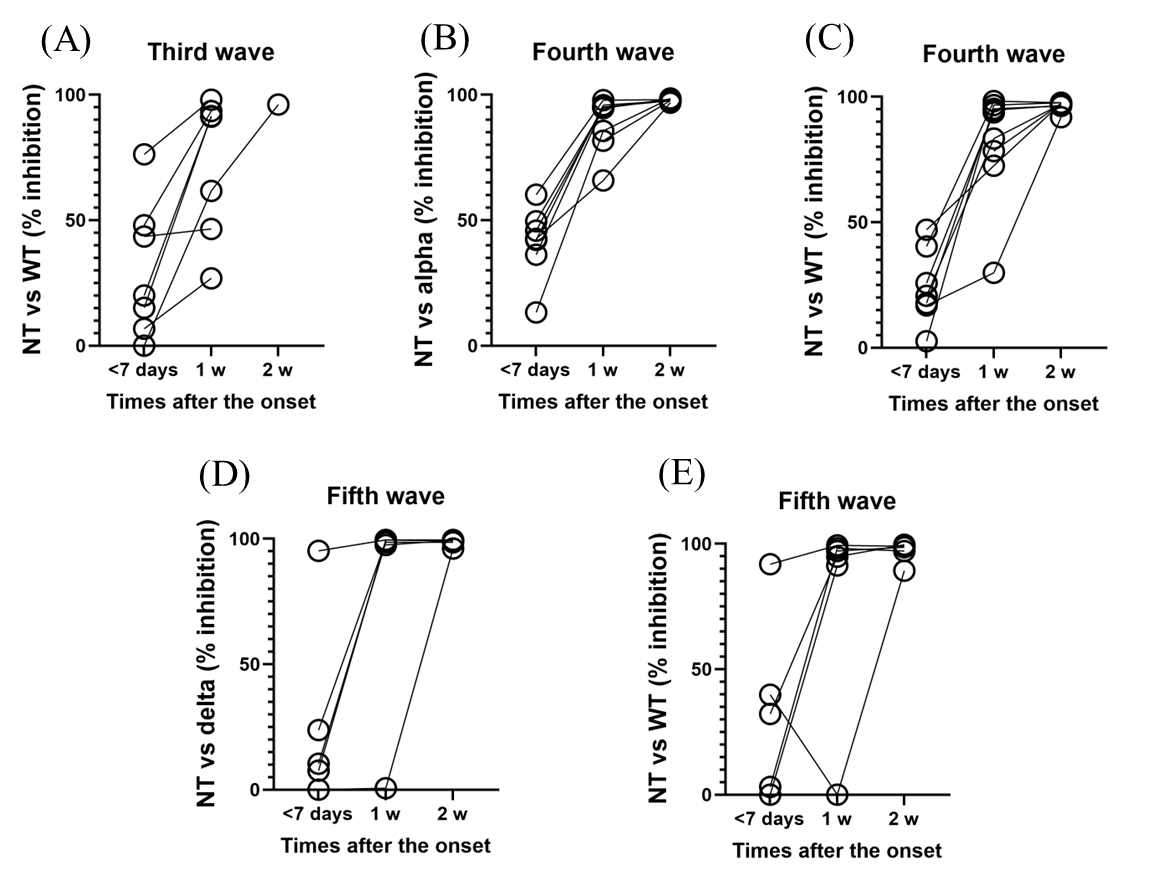


Time-dependent change of serum neutralizing activities (NT; % inhibition) in patients with SARS-CoV-2 infection. NT against each SARS-CoV-2 variant was evaluated at hospital admission (within five days after symptom onset), then 1 week and 2 weeks after. The number of patients included in this analysis was as follows; 7 patients enrolled in the third pandemic wave, 9 patients enrolled in the fourth pandemic wave and 6 patients enrolled in the fifth pandemic wave. The follow-up for NT until 2 weeks after hospital admission was available with only 1 patient enrolled in third pandemic wave, since the large population discharged within a week after admission. Whilst, the follow-up evaluation for NT were available with 8 patients enrolled in the fourth pandemic wave and with 5 patients enrolled in the fifth pandemic wave. NT against the wild-type strain or infected variant was elevated to >90% until 2 weeks for all the patients whose serums were available.

**Figure Supple 2**


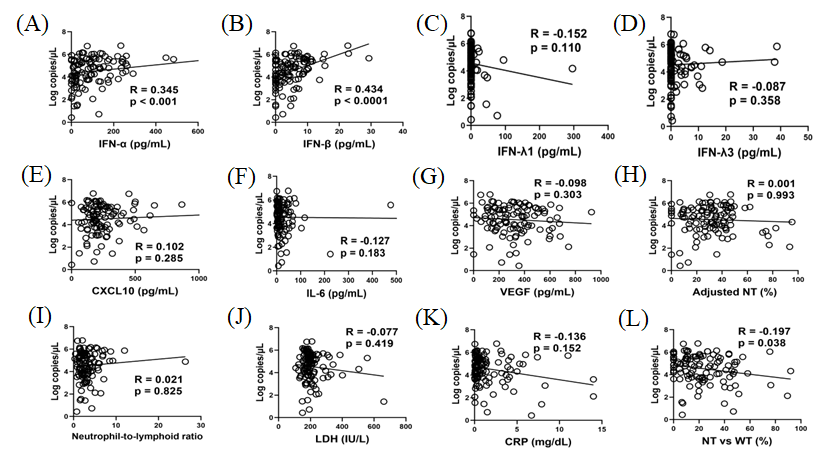


Correlations between serum biomarker levels and SARS-CoV-2 viral load in nasal swab specimen with COVID-19 patients at hospital admission (within five days after symptom onset); (A) IFN-α, (B) IFN-β, (C) IFN-λ1, (D) IFN-λ3, (E) CXCL10, (F) IL-6, (G) VEGF, (H) Adjusted NT, (I) NLR, (J) LDH, (K) CRP and (L) NT vs WT. Spearman correlation test was used, and Spearman correlation coefficient is shown. Corresponding logarithmic trendlines are shown. Several values were not plotted because measured values were remarkably high; 2 values for IFN-α (1200 pg/mL and 1040 pg/mL), 1 value for IL-6 (1708 pg/mL), 4 values for CXCL10 (>2000 pg/mL). CRP, C-reactive protein; CXCL10, C-X-C motif chemokine ligand 10; IFN, interferon; IL, interleukin; LDH, lactate dehydrogenase; NLR, neutrophil-to-lymphocyte ratio; NT, neutralizing activities (% inhibition); NT vs WT, neutralizing activities against the wild-type strain; VEGF, vascular endothelial growth factor.

**Figure Supple 3**


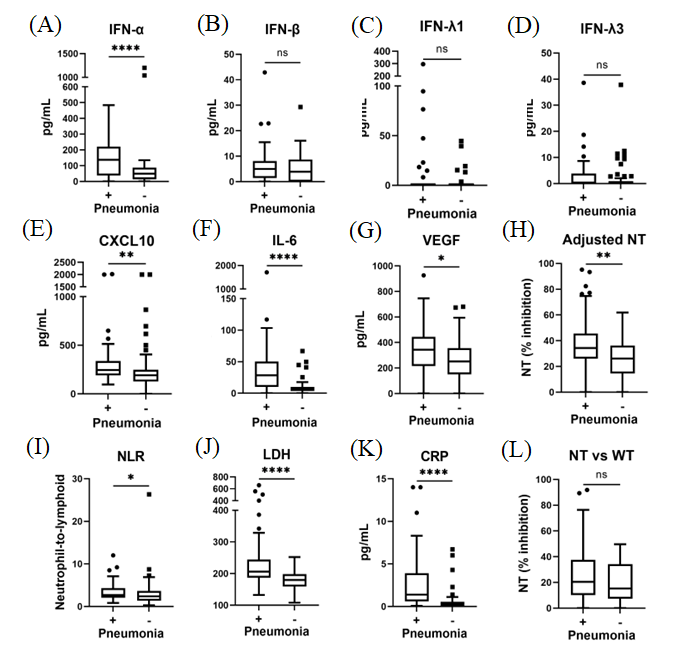


Serum biomarker levels in the early phase of SARS-CoV-2 infection and associations with the presence of pneumonia; (A) IFN-α, (B) IFN-β, (C) IFN-λ1, (D) IFN-λ3, (E) CXCL10, (F) IL-6, (G) VEGF, (H) Adjusted NT, (I) NLR, (J) LDH, (K) CRP and (L) NT vs WT. Each level was evaluated at hospital admission (within five days after symptom onset). Data are presented as Tukey box-plots and individual values. Nonparametric Mann-Whitney test was used to compare values between groups. ^＊^; p<0.05. ^＊＊^; p<0.005. ^＊＊＊^; p<0.001. ^＊＊＊＊^; p<0.0001. NT, neutralizing activities (% inhibition); NT vs WT, neutralizing activities against the wild-type strain.

**Figure Supple 4**


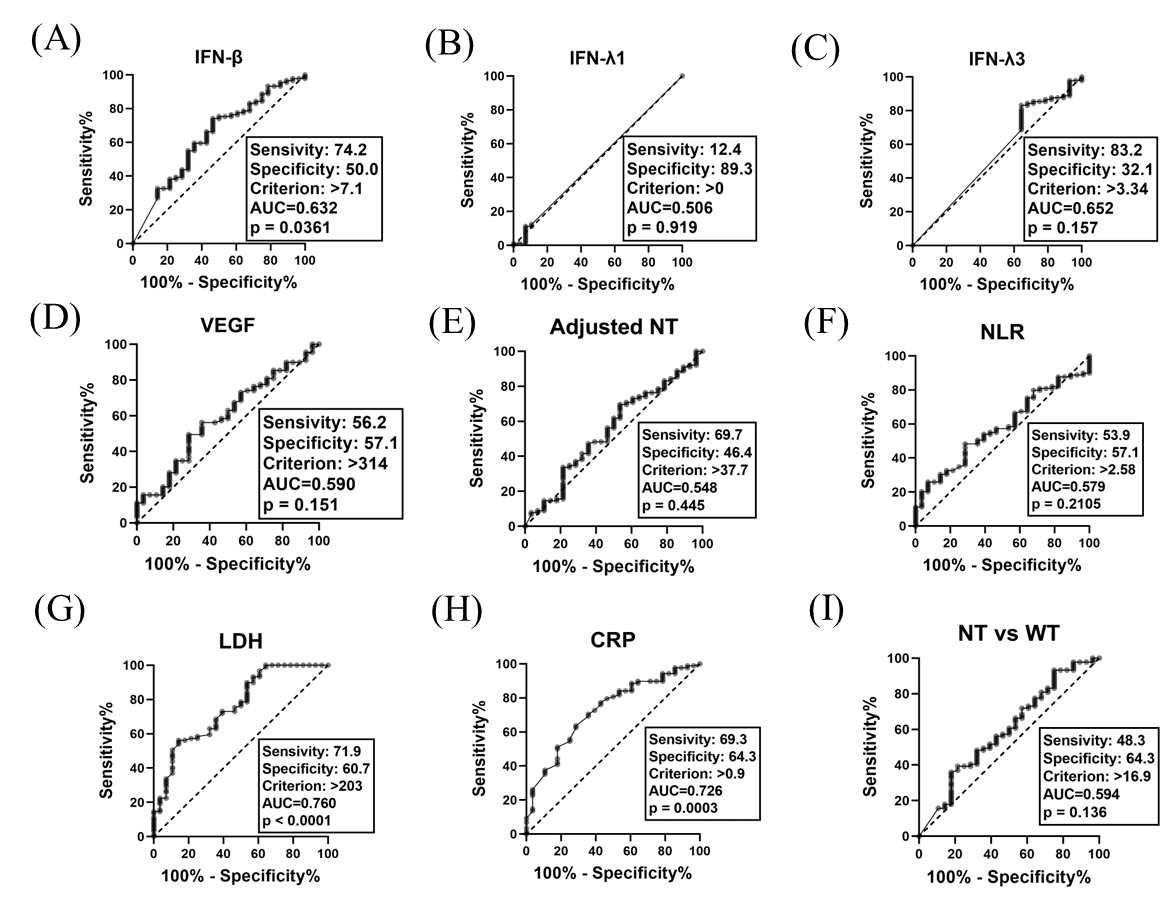


Fig S4. ROC curves and AUCs for biomarker levels in patients with SARS-CoV-2 infections in regard to diagnostic values indicating respiratory failure: (**A**) IFN-β, (**B**) IFN-λ1, (**C**) IFN-λ3, (**D**) VEGF, (**E**) adjusted NT, (**F**) NLR, (**G**) LDH, (**H**) CRP, and (**I**) NT vs WT. AUC, area under ROC curve; NT, neutralizing activities (% inhibition); NT vs WT, neutralizing activities against the wild-type strain; ROC, receiver operating characteristic.
